# Disrupted Global Brain Dynamics in Adolescents With Comorbid Anxiety and Depression: Neural Mechanisms and Classification Based on EEG Microstates

**DOI:** 10.1155/da/4012249

**Published:** 2026-07-15

**Authors:** Shangfeng Han, Yaohui Lin, Jie Gao, Yufu Wang, Bingjin Zheng, Yuejia Luo, Pengfei Xu

**Affiliations:** ^1^ Department of Psychology and Center for Brain and Cognitive Sciences, School of Education, Guangzhou University, Guangzhou, China, gzhu.edu.cn; ^2^ Dujiangyan Special Crew Sanatorium of PLA Air Force, Dujiangyan, Sichuan, China; ^3^ School of Psychology, Sichuan Center of Applied Psychology, Chengdu Medical College, Chengdu, China, cmc.edu.cn; ^4^ Beijing Key Laboratory of Applied Experimental Psychology, National Demonstration Center for Experimental Psychology Education (BNU), Faculty of Psychology, Beijing Normal University, Beijing, China, bnu.edu.cn; ^5^ School of Psychology, Shenzhen University, Shenzhen, China, szu.edu.cn; ^6^ Institute for Neuropsychological Rehabilitation, University of Health and Rehabilitation Sciences, Qingdao, China; ^7^ School of Psychology, South China Normal University, Guangzhou, China, scnu.edu.cn; ^8^ Faculty of Health and Wellness, City University of Macau, Macau, China, cityu.edu.mo

**Keywords:** adolescents, anxiety–depression comorbidity, classification, functional connectivity, microstate, time-frequency

## Abstract

**Background:**

Adolescents with comorbid anxiety and depression (ACAD) represent a significant mental health challenge that necessitates the identification of objective neurobiological markers for accurate diagnosis and intervention. Previous electroencephalography (EEG) studies have often relied on single‐domain or single‐feature analyses, which may fail to capture the multidimensional nature of the underlying neuropathology. This study aimed to characterize neurophysiological alterations in adolescents with ACAD using a multidimensional EEG analytical framework integrating spectral power, functional connectivity (FC), and microstate (MS) dynamics.

**Methods:**

Forty adolescents with ACAD and 42 healthy controls (HCs) were included. Resting‐state EEG data were analyzed to extract spectral power, weighted phase lag index‐based FC, and MS parameters. Three types of support vector machine (SVM) classification were implemented, including single‐feature SVM analysis, multivariate SVM (MV‐SVM) without principal component (PC) analysis (PCA), and PCA‐based SVM, to distinguish the independent discriminative value of individual features from the effects of multivariate feature integration and PCA‐based dimensionality reduction.

**Results:**

Adolescents with ACAD showed marginally reduced delta power, altered alpha‐band FC, and disrupted MS dynamics, including altered MS class expression and abnormal transition probabilities. Among individual MS features, the transition probability from MS B to MS C showed the highest independent discriminative value, achieving an accuracy of 75% and an area under the receiver operating characteristic (ROC) curve (AUC) of 0.86. Multivariate integration of all MS parameters substantially improved classification performance, with the MV‐SVM achieving an accuracy of 0.95 and an AUC of 0.98. The PCA –SVM model showed comparable performance, with an accuracy of 0.94 and an AUC of 0.98, while reducing correlated MS features to three PCs explaining more than 90% of the variance.

**Conclusion:**

These findings suggest that adolescent ACAD is characterized by global dynamic network dysregulation rather than isolated local abnormalities. Integrated MS dynamics, particularly when modeled in a multivariate framework, may serve as candidate biomarkers for distinguishing adolescents with ACAD from HCs. This multidimensional EEG approach may contribute to early detection, risk stratification, and targeted intervention in adolescent mental health.

## 1. Introduction

Adolescence is a pivotal neurodevelopmental window marked by rapid maturation of cognitive and affective systems that support adaptation to complex social and environmental demands [1]. This same period also carries the highest incidence of common mental disorders, with the peak age of onset around 14.5 years and up to 34% of adolescents (10–19 years) at risk for clinical depression [2]. In addition to depression, anxiety is highly prevalent during adolescence, with meta‐analytic evidence indicating that the prevalence of clinically elevated anxiety symptoms reaches 20.5%, while the pooled 12‐month prevalence of anxiety disorders is approximately 9.0% [3]. Of particular clinical significance is the high comorbidity between anxiety and depression during adolescence, with evidence indicating that the co‐occurrence rate can be as high as 31.6% in adolescents [4, 5]. Moreover, adolescents with comorbid anxiety and depression (ACAD) exhibit more severe symptomatology, poorer treatment response, and greater functional impairment compared to those with single disorders [5–8]. The complexity of this comorbid presentation poses significant challenges for accurate diagnosis and effective intervention, highlighting the urgent need for objective biomarkers that can reliably distinguish ACAD from healthy controls (HCs).

An increasing body of research has focused on identifying electroencephalography (EEG)‐based electrophysiological biomarkers for ACAD [9–11]. From a neural oscillation perspective, patients with major depressive disorder (MDD) comorbid with anxiety have been found to exhibit globally elevated beta power, consistent with the hypothesis of heightened cortical arousal [12]. Moreover, increased frontal alpha asymmetry has been reported in MDD with comorbid anxiety, with this pattern remaining stable over a 5‐year period spanning adolescence to early adulthood [13]. At the functional connectivity (FC) level, alpha‐band connectivity is positively correlated with both depression and anxiety symptom severity, constituting one of the most informative predictors of symptom scores [14]. Depression has also been associated with increased alpha‐band power and connectivity [9] as well as elevated beta‐band connectivity [15]. In contrast, anxiety disorders are often characterized by decreased connectivity spanning theta through gamma frequencies [16, 17]. Collectively, these findings indicate that alterations in both time –frequency dynamics and FC are relevant to the pathophysiology of anxiety and depression. However, the inconsistencies across studies highlight the need for systematic, comparative investigations to validate robust EEG biomarkers, particularly in adolescent populations.

Recent advancements in EEG have suggested the potential of microstates (MSs) as novel biological markers for mental disorders. EEG MSs, defined as brief stable patterns in EEG signals, represent dynamic changes in large‐scale brain networks and provide insights into the transient reconfiguration of functional networks [18]. These MSs are typically categorized into four classes (A, B, C, and D). Four key temporal parameters —occurrence, duration, coverage, and transition probabilities (TPs) — prove useful for investigating psychiatric characteristics, offering new perspectives for diagnosis and monitoring [19–21]. Notably, abnormalities in MS dynamics have been observed across various mental disorders, including anxiety and depression. For instance, changes in MS A and MS B were reported, and meta‐analytic findings reveal a significant increase in the duration of MS A in depression [20] and the increased prevalence of MS A and B in anxiety, suggesting their potential as specific markers [20, 22]. Other studies found social anxiety [23] and depression [24] have an increased presence of MS C and decreased presence of MS D compared to HC. Studies of microstate TPs of microstates have found disrupted dynamic switching among the four microstates in anxiety and depression [23, 25, 26]. These findings underscore the potential of EEG MSs as a promising tool for understanding the neural mechanisms underlying anxiety and depression.

Numerous studies have successfully identified neurophysiological indicators that demonstrate abnormalities of the MS in psychiatric populations [22, 27]. These MS alterations exhibit remarkably similar patterns across diverse psychiatric conditions, thereby undermining their potential as disorder‐specific biomarkers. This specificity challenge is exemplified by the widespread occurrence of comparable MS abnormalities across multiple psychiatric disorders. The well‐documented alterations in MS C and MS D, initially identified in schizophrenia, have been subsequently replicated in Parkinson’s disease, social anxiety, and depression [23, 24, 28, 29]. Similarly, pathological changes in MS A and MS B have been reported across various conditions [20, 22], suggesting that individual MS parameters may reflect shared neurobiological vulnerabilities rather than specific pathophysiology. Furthermore, the observation that all MS indicators (A–D) exhibit abnormalities in previous studies [18, 20, 22] poses a significant challenge in identifying the optimal biological markers for specific diseases.

The previous research landscape is mainly constrained by the predominant reliance on univariate analytical approaches. Existing studies typically use single‐feature analysis strategies, focusing exclusively on independent statistical comparisons of individual MS parameters between patients and controls. This reductionist approach fails to capture the complex, multidimensional nature of neural dysfunction in psychiatric disorders and neglects the potential synergistic information embedded within integrated feature combinations. Consequently, the field lacks a comprehensive understanding of which EEG feature combinations provide optimal discriminatory power for precise classification of specific psychiatric populations, particularly ACAD. This methodological gap represents a critical barrier to establishing clinically viable neurophysiological markers and severely impedes the translational application of EEG biomarkers from research settings to clinical practice.

The present study adopts a comprehensive multidimensional analytical framework designed to systematically characterize the complete electrophysiological signature of ACAD. Rather than examining isolated parameters, our approach systematically integrates all the parameters in multidimensional EEG signals, including spectral power, FC, and MS, to capture neural dysfunction complexity. For classification, we employ support vector machine (SVM) algorithms, a method particularly well‐suited to the analytical challenges inherent in EEG data. EEG signals are high ‐dimensional, nonstationary, and typically derived from relatively small clinical samples —conditions under which SVM demonstrates robust generalization performance by virtue of its structural risk minimization framework [30]. Furthermore, the kernel‐based architecture of SVM enables effective modeling of nonlinear decision boundaries, which are characteristic of the complex relationships between neurophysiological features and clinical phenotypes [31]. This data‐driven approach enables systematic screening of multivariate feature combinations while accounting for the inherent nonlinearity and high dimensionality of the EEG signal space.

The primary objectives of this study are twofold: (1) to characterize the neurophysiological signature of ACAD through multiscale EEG analyses encompassing neural oscillations, FC, and MS dynamics and (2) to identify the most discriminative feature combinations that maximize classification accuracy. Our findings are anticipated to provide objective, quantifiable indices for the precise assessment of adolescent affective disorders and inform the development of personalized, targeted interventions that address the specific neurobiological alterations underlying comorbid presentations.

## 2. Methods and Materials

### 2.1. Participants

Forty adolescents (15 female adolescents and 25 male adolescents) with clinically significant levels of both anxiety and depression were identified through clinical screening from an initial cohort of 120 hospitalized youth diagnosed by DSM‐5 with comorbid anxiety and depressive disorders (age 15.05 ± 1.92 years (mean ± SD), range 12–18 years) in the disorder group. The study participants were selected based on strict clinical cutoff scores, with all subjects meeting the following criteria: generalized anxiety disorder (GAD) scores above 14 points and patient health questionnaire (PHQ) scores above 15 points. The HC group included 42 adolescents (12 female and 30 male), aged 12–19 years (mean ± SD *=* 15.55 ± 1.94). No significant difference was found in age between the patients and HC, *t*(80) = 1.17, *p* = 0.25. But the gender showed significant differences between two groups, *χ* ^2^ = 19.23, *p* < 0.001. Therefore, we used analysis of covariance ( ANCOVA ) with gender as a covariate in the following analyses. In the ACAD group, the proportion of patients on medication was 71.80%, and the duration of illness was 13.67 ± 12.62 months (Table 1). All participants were right ‐handed, with normal or corrected vision; they also provided informed assent. The study was approved by the Ethics Committee of the authors’ university (number 2023052). This study followed the STROBE reporting guideline for observational studies.

**Table 1:** Demographic and clinical characteristics of participants.

Characteristics	ACAD group (*N* = 40)	HC group (*N* = 42)	*t*/*χ* ^2^	*p*‐Value
Gender	15 female, 25 male	12 female, 30 male	*χ* ^2^ = 19.23	*p* < 0.001
Age, *M* ± SD	15.05 ± 1.92	15.55 ± 1.94	*t*(80) = 1.17	*p* = 0.25
GAD, *M* ± SD	18.53 ± 2.14	—	—	—
PHQ, *M* ± SD	22.24 ± 3.56	—	—	—
Medication rate, *N* (%)	71.80%	—	—	—
Illness duration (months), *M* ± SD	13.67 ± 12.62	—	—	—

Abbreviations: ACAD, adolescents with comorbid anxiety and depression; GAD, generalized anxiety disorder scale; HCs, healthy controls; PHQ, patient health questionnaire; SD, standard deviation.

### 2.2. Questionnaires

The severity of participants’ anxiety symptoms was assessed with the 7‐item GAD scale (GAD‐7) [32]. This widely used self‐report questionnaire consists of seven items that evaluate the severity of anxiety symptoms over the past 2 weeks. Each item was scored on a 4‐point Likert scale, ranging from 0 (“not at all”) to 3 (“nearly every day”). Scores are interpreted as follows: 0–4 indicates minimal anxiety, 5–9 indicates mild anxiety, 10–14 indicates moderate anxiety, and 15–21 indicates severe anxiety. GAD‐7 has demonstrated strong psychometric properties, including high internal consistency (Cronbach’s alpha typically around 0.89–0.92) and good test –retest reliability (intraclass correlation coefficient of 0.83) [32].

Depressive symptom severity was assessed with the 9‐item PHQ (PHQ‐9) [33]. The PHQ‐9 is a brief self‐report instrument that operationalizes the nine DSM‐5 criteria for MDD. Respondents indicate how often they have been bothered by each symptom during the preceding 2 weeks (e. g., “little interest or pleasure in doing things”) on a 4‐point Likert scale ranging from 0 (“not at all”) to 3 (“nearly every day”). Item scores were summed to yield a total score between 0 and 27, with higher scores indicating more severe depressive symptoms. Standard cut‐points are 5 (mild), 10 (moderate), 15 (moderately severe), and 20 (severe) depression [33]. The final item addressing self‐harm thoughts was simultaneously scored and treated as a clinical safety screen. In the present sample, PHQ‐9 exhibited excellent internal consistency (Cronbach’s *α* = 0.89). A single‐factor confirmatory factor analysis supported its unidimensional structure (comparative fit index = 0.97 and root mean square error of approximation = 0.06).

### 2.3. Design

To examine alterations in ACAD, a 2 (Group: patients and controls) × 4 (State: MS A, MS B, MS C, and MS D) mixed factorial design was employed. For the FC and time –frequency analyses, a 2 (Group: patients and controls) × 4 (FC of Frequency band: delta, theta, alpha, beta) mixed factorial design was employed, and a 2 (group: patients and controls) × 4 (Frequency band: delta, theta, alpha, beta) mixed factorial design was performed for the time –frequency analysis. In these designs, group served as a between‐subjects factor, and another factor was treated as a within‐subjects factor. Gender was included as a covariate in all analyses. Repeated‐measures ANOVAs were conducted without assuming sphericity, and the Geisser –Greenhouse correction was applied to adjust for violations of this assumption. To control for familywise type I error arising from multiple pairwise comparisons, Holm correction [34] was applied to all post hoc pairwise comparisons within each analytical domain using JASP (Version 0.19.3.0).

### 2.4. EEG Data Recording and Preprocessing

Resting‐state EEG was recorded while participants sat quietly with their eyes closed for 3 min. Signals were acquired with a 64‐channel actiCHamp system (Brain Products GmbH, Gilching, Germany) at a sampling rate of 500 Hz. Ag/AgCl electrodes embedded in a 64‐channel cap were positioned according to the international 10–20 system and referenced online to FCz. The electrode impedance was maintained below 5 kΩ throughout the recording.

The EEG data were imported into EEGLAB [35] in MATLAB (R2022b). The preprocessing procedure is as follows: (1) data were filtered off‐line by a bandpass filter of 0.1–30 Hz with a Butterworth IIR filter after a notch filter for 50 Hz line noise [36]. (2) Through visual inspection, bad channels that contained excessive noise or artifacts were marked and excluded for the next step of independent component analysis (ICA). ICA based on the extended infomax algorithm [37] was used to remove the components related to eye movements, blinks, head movement, electro cardio, and other types of artifacts [38, 39]. (3) The marked bad channels were interpolated using spherical interpolation [40]. (4) The artifact‐free data underwent average re‐referencing. For the MS analysis specifically, the artifact‐free EEG data were additionally band‐pass‐filtered between 2 and 20 Hz using a Butterworth IIR filter [18]. This MS‐specific filtering was not applied to the datasets utilized for time –frequency and FC analyses. Finally, the preprocessed data underwent MS analysis for each participant.

### 2.5. MS Analysis

To investigate the temporal dynamics at rest, we performed EEG MS analysis on the preprocessed, artifact‐free continuous data using the MS toolbox for EEGLAB [41]. The first step is the segmentation and identification of MS maps. Specifically, the global field power (GFP) was calculated for each participant’s data. GFP represents the spatial standard deviation of the electric potential across all electrodes at a given moment in time, providing a measure of the overall strength of the scalp potential field. The EEG topographies at the peaks of the GFP signal were extracted for clustering as these time points exhibit the highest signal‐to‐noise ratio. The extracted topographies from all participants were then submitted to a modified *k*‐ means clustering algorithm to identify the most dominant and stable MS maps. While some methodological guidelines recommend determining the optimal number of MS clusters using data‐driven optimization criteria [18], we chose to set the number of clusters to four a priori. This decision was primarily driven by the fundamental need for cross‐study comparability and clinical interpretability. As highlighted in a recent large‐scale meta‐analysis [22], MS temporal parameters (e. g., duration, occurrence, and coverage) are inherently dependent on the number of clusters used, rendering cross‐study comparisons unviable when variable class numbers are adopted. Furthermore, the canonical four MSs (A, B, C, and D) remain the most consistently observable and widely accepted topographies, typically explaining 65%–80% of the global variance in resting‐state EEG and linking directly to well‐established functional resting‐state networks [18, 42, 43]. Therefore, fixing the number of classes to four allows our findings in ACAD to be reliably compared with those in existing neurophysiological models and prior clinical cohorts. This procedure resulted in a set of group‐level template maps representing the most common scalp topographies across all participants. The second step is back‐fitting and the calculation of temporal parameters. The obtained group‐level template maps were then fitted back to the continuous EEG data of each individual participant using spatial correlation. At each time point, the original EEG topography was labeled with the MS map with which it shared the highest spatial correlation. This process segments the entire EEG recording into a continuous sequence of microstates. From this symbolic sequence, we calculated the following standard temporal parameters for each microstate class for each participant:

1. Duration (the mean duration of one MS that remains quasi‐stable) is generally believed to reflect the stability of the brain state.
2. Coverage (the percentage of time one MS holds in the whole recording) is interpreted as a relative time coverage of the neural generator of one MS relative to other neural generators.
3. Occurrence (the number of times a single MS occurs per second) reflects the trend that the potential neural generator is activated.
4. GFP reflects the strength or coordination of neurons in the basic neural generator [18, 42].
5. TPs: The probability of transitioning from any given MS class to any other class. This was calculated as the observed transition frequency divided by the total number of transitions away from the source MS, resulting in a transition matrix for each participant.

### 2.6. Time‐Frequency Analysis

Time‐frequency analysis was performed to investigate the neural oscillation dynamics of the EEG signals using the FieldTrip toolbox [44] implemented in MATLAB (R2022b). For each subject, the continuous preprocessed EEG data was first segmented into nonoverlapping 2‐s epochs. Subsequently, the power spectral density (PSD) was estimated for each epoch using a fast Fourier transform (FFT) based on a multitaper method (mtmfft) with a single Hanning taper. This analysis was conducted for frequencies ranging from 1 to 30 Hz. This procedure resulted in a time –frequency representation (TFR) for each subject, where the consecutive 2‐s epochs constituted the time axis. To obtain a group‐level representation, the individual subjects’ TFRs were averaged to compute a grand‐average TFR across all participants.

For visualization purposes, two analyses were conducted on the grand‐average data. First, to examine the spatial distribution of neural oscillations, power was averaged within four canonical frequency bands: delta (1–4 Hz), theta (4–8 Hz), alpha (8–13 Hz), and beta (13–30 Hz). Second, to visualize the overall time‐frequency dynamics, the power from the grand‐average TFR was first averaged across all channels. The resulting power values were converted to a decibel (dB) scale (10^∗^log10(power)) and smoothed using a Gaussian filter to enhance clarity. This produced a final time‐frequency plot showing power changes across time and frequency, averaged over the entire scalp. The processed time‐frequency analysis data was then used for FC analysis.

### 2.7. FC Analysis

We calculated the FC between all pairs of EEG channels using the debiased weighted phase lag index (wPLI) (method = “wpli_debiased”). The wPLI is a measure of phase synchronization that is robust against the effects of volume conduction and less sensitive to noise and sample size bias [45]. This procedure yielded a frequency‐resolved connectivity matrix (channel × channel × frequency) for each subject. To obtain band‐specific connectivity metrics, the wPLI values were then averaged across the frequencies within delta, theta, alpha, and beta bands. The selection of these four canonical frequency bands was guided by both neurobiological relevance and the inherent technical characteristics of scalp EEG. From a neurobiological standpoint, delta and theta oscillations have been consistently implicated in emotional regulation, motivational processing, and salience detection —functions known to be dysregulated in anxiety and depressive disorders [46]. Alpha‐band activity serves as a critical index of cortical inhibition, and its connectivity has been established as a sensitive marker of large‐scale network integration in adolescence [47, 48]. Beta‐band oscillations are associated with active cognitive processing and affective regulation and often altered in mood disorder populations [16]. Furthermore, higher‐frequency oscillations in the gamma range (>30 Hz) were deliberately excluded from the present analysis. Although gamma activity carries functional relevance, scalp EEG recordings are fundamentally limited in their capacity to capture gamma‐band signals with sufficient fidelity. Due to the substantial attenuation of high‐frequency neural signals by the skull and scalp tissues, as well as the high susceptibility of this frequency range to severe contamination by electromyographic (EMG) muscle artifacts, the signal‐to‐noise ratio of gamma‐band activity in scalp EEG is considerably lower than that of lower‐frequency oscillations [49, 50]. This specific band selection therefore reflects both the neurophysiological focus on ACAD and established methodological conventions for ensuring signal reliability. This process resulted in a single adjacency matrix (channel × channel) for each subject within each of the specified frequency bands.

### 2.8. Classification and Statistical Comparison

To classify ACAD and HC, we employed SVM classifiers on EEG‐derived features of MSs, FC across frequency bands, and time‐frequency signals. These features were extracted from preprocessed resting‐state EEG data, as detailed in the preceding sections. Classification was performed independently for each feature type to evaluate their individual discriminatory power. Additionally, principal component (PC) analysis (PCA) was applied for dimensionality reduction prior to SVM to assess potential improvements in performance due to reduced feature complexity. Classification efficacy was compared across the three feature types using metrics, including accuracy, sensitivity, specificity, and area under the receiver operating characteristic (ROC) curve (AUC). The details are as follows:

### 2.9. Feature Extraction

This study utilized an SVM algorithm to differentiate between the ACAD group and the HC group. Three categories of discriminative features were systematically incorporated into the model: (1) MS parameters, including mean duration, occurrence rates, coverage, GFP, and TPs among four MSs; (2) FC metrics, quantified by wPLI and averaged across delta, theta, alpha, and beta frequency bands; and (3) time –frequency characteristics, derived by segmenting preprocessed EEG into 2‐second epochs and computing spectral power (1–30 Hz) using the multitaper method (mtmfft). These features were meticulously selected to comprehensively capture both the temporal dynamics and spectral properties of brain activity, thereby providing a robust basis for group differentiation.

### 2.10. SVM Classification

To evaluate the discriminative power of individual EEG features for classifying the ACAD group from the HC group, we conducted a machine learning analysis by using the caret package implemented in R (version 4.5.1), which provides a unified framework for model training and evaluation.

A SVM with a nonlinear radial basis function (RBF) kernel (svmRadial) was chosen as the classifier. The machine learning pipeline was structured into three conditions (single‐feature SVM, multivariate SVM [MV‐SVM], and PCA‐based SVM) specifically designed to disentangle the independent contributions of multivariate feature integration and PCA‐based dimensionality reduction to classification performance. Single‐feature SVM was designed solely to quantify the independent discriminative capacity of each individual EEG feature. By training a separate SVM model on each single feature in isolation, single‐feature SVM provides a filter‐based feature importance estimate that identifies which feature domain contributes the most informative signal to subsequent multivariate modeling. The performance of each model was assessed using a robust repeated 10‐fold cross‐validation scheme with 100 repetitions. This procedure involves splitting the data into 10 folds, training the model on nine folds and testing on the remaining one, and repeating this process 100 times to obtain a stable estimate of performance. Within each cross‐validation loop, features were standardized by centering and scaling (preProcess = c (“center,” “scale”)) to prevent data leakage and ensure comparability. Specifically, prior to classifier training, feature values were normalized through centering and scaling. To strictly prevent data leakage, all normalization procedures were performed within each cross‐validation fold: the mean and standard deviation used for centering and scaling were computed solely from the training partition of each fold and subsequently applied to the corresponding held‐out test partition. No information from the test fold was used at any stage of preprocessing or model training. This within‐fold normalization strategy ensures that performance estimates derived from the cross‐validation procedure are unbiased and reflect true out‐of‐sample generalization ability [51, 52]. To quantify the relative contribution of each individual feature to classification performance, variable importance was assessed using the varImp() function implemented in the caret package. For SVM classifiers with an RBF kernel, this function computes a filter‐based variable importance score for each feature by calculating the AUC derived from the feature’s univariate association with the binary group outcome (ACAD vs. HC) across all cross‐validation folds. Features are subsequently ranked in descending order of their individual AUC scores, such that the feature with the highest AUC is identified as the most contributory to classification.

To optimize the model performance, a grid search was employed for hyperparameter tuning. The cost parameter C (tested values: 0.1, 1, and 10) and the RBF kernel parameter sigma (tested values: 0.01, 0.05, and 0.1) were systematically evaluated. The optimal combination of hyperparameters was selected based on the model that yielded the highest AUC, which was set as the primary performance metric for tuning (metric = “ROC”).

The classification efficacy of the best‐tuned model for each feature was quantified by its accuracy, sensitivity, specificity, and AUC. The mean and standard deviation of these metrics were calculated across all 1000 folds (10 folds × 100 repeats) to provide a comprehensive and reliable assessment of each feature’s predictive value. Critically, stratified sampling was applied throughout all cross‐validation folds. Specifically, the trainControl function in the caret package implements stratified splitting by default, ensuring that the class distribution of the full dataset (ACAD vs. HC) was proportionally preserved in both the training and test sets of every fold across all 100 repetitions [53]. Although the overall case‐to‐control ratio in the present study was approximately 1:1, this stratification strategy was retained as a methodological safeguard to prevent any incidental class imbalance that may arise from random partitioning in small clinical samples, thereby ensuring unbiased and stable performance estimates across all folds. This process was repeated independently for MS features, each frequency band’s FC features, and time‐frequency features.

### 2.11. MV‐SVM Without PCA

All MS features were simultaneously entered as inputs to a single SVM classifier with within‐fold standardization (centering and scaling) but without PCA (preProcess = c (“center,” “scale”)). All other aspects of the pipeline —SVM kernel (svmRadial), hyperparameter grid search, and repeated 10‐fold cross‐validation with 100 repetitions —were identical to single‐feature SVM. This condition isolates the contribution of multivariate feature representation independent of any dimensionality reduction. The comparison between the single‐feature SVM and the MV‐SVM analysis directly addresses whether integrating multiple features improves classification performance beyond that achieved by individual features alone.

### 2.12. PCA –SVM Classification

We developed a MV‐SVM model to capture the synergistic information embedded within feature combinations, which represents the core analytical novelty of this study. To address the challenges inherent in high‐dimensional EEG data, such as multicollinearity, noise, and computational inefficiency, PCA was employed as a critical preprocessing step prior to classification. While SVM algorithms are theoretically capable of handling multiple features simultaneously, directly training a MV‐SVM on all extracted EEG parameters in our relatively small clinical sample would introduce a severe risk of overfitting and the “curse of dimensionality” [54]. PCA is essential in this context because multidimensional EEG features often exhibit strong correlations, leading to redundant information that can degrade model performance and increase overfitting risk [55]. By transforming the original features into a reduced set of uncorrelated PCs, PCA captures the majority of the data’s variance in fewer dimensions, thereby enhancing model interpretability, reducing noise, and improving computational efficiency without a significant loss of information. This is particularly advantageous for SVM‐based classification in neuroimaging studies, where it mitigates the “curse of dimensionality” and promotes better generalization, especially with limited sample sizes typical in clinical EEG datasets.

Prior to PCA, all features were standardized (centered and scaled) to ensure that variables with larger variances did not disproportionately influence the analysis. The optimal number of PCs to retain for subsequent classification was determined through a two‐step visual analysis: (1) identifying the “elbow point” in the scree plot, which indicates diminishing returns in explained variance and (2) examining the cumulative variance plot to ensure the selected PCs accounted for a substantial portion of the total data variance (>90%). Based on this analysis, hree PCs were retained for each feature domain as they collectively accounted for more than 90% of the total variance in each respective feature set.

In the following, SVM classification, including feature standardization and the PCA transformation, was embedded within each fold of the cross‐validation procedure (preProcess = c (“center,” “scale,” “pca”)). This nested approach prevents data leakage by ensuring that the PCA model is built using only the training data for each fold, providing a rigorous assessment of the model’s performance on unseen data. The comparison between MV‐SVM and PCA‐based SVM directly addresses whether PCA‐based dimensionality reduction provides additional classification benefit beyond multivariate feature combination alone, holding the feature set constant across both conditions. Other steps align with the preceding SVM processing workflow.

### 2.13. Permutation Testing

To assess whether the observed classification performance was statistically distinguishable from chance level, label‐permutation tests were conducted for both the best univariate SVM model and the PCA –SVM model. In each of the 1000 iterations, class labels were randomly shuffled, while all other pipeline components (within‐fold standardization, PCA transformation, hyperparameter grid search, and repeated 10‐fold cross‐validation) remained identical to the original analysis. The permutation‐based *p*‐ value was calculated as (*k* + 1)/(*n* + 1), where *k* denotes the number of permuted iterations yielding a metric equal to or exceeding the observed value and *n* is the total number of valid permutations [56].

## 3. Results

### 3.1. MS Results

**Figure 1:**
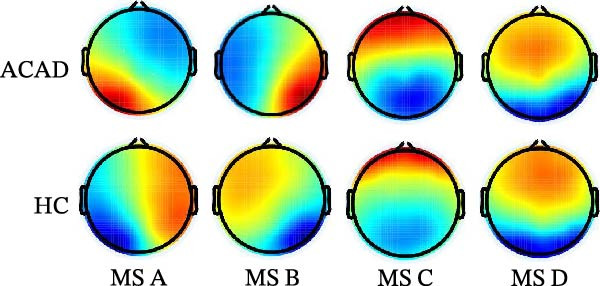
Topographic distributions of microstate (MS) patterns in adolescents with comorbid anxiety and depression (ACAD) and healthy control (HC) groups.

**Figure 2:**
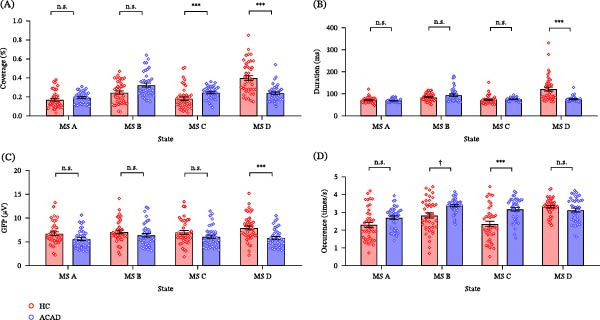
Comparison of microstate parameters between adolescents with comorbid anxiety and depression (ACAD) and HC (healthy control) groups across four microstate classes (MS A–D). Four parts display different microstate metrics: (A) coverage (%), representing the proportion of time each microstate is present; (B) duration (ms), indicating the average time duration of each microstate; (C) GFP (μV), showing the global field power associated with each microstate; and (D) occurrence (times/s), reflecting the frequency of microstate transitions per second. Individual data points are overlaid on bar graphs showing mean ± SEM. Statistical comparisons between groups are indicated above each comparison: n.s., not significant; † = 0.05 < *p* < 0.1;  ^∗∗∗^ *p* < 0.001.

Through MS analysis, we identified four classic MSs, the topographical maps of which are presented in Figure 1. For MS coverage, a significant Group × State interaction was observed, *F*(3, 237) = 13.30, *p* < 0.001, *η* ^2^ = 0.14. Simple effects analysis revealed that MS C coverage was significantly higher in the ACAD group compared to the HC group (*p* < 0.001), whereas MS D coverage was significantly lower in the ACAD group (*p* < 0.001). No significant group differences were found for MS A or MS B (ps > 0.17). There was also no significant main effect of State, *F*(3, 237) = 1.90, *p* = 0.13, and no significant main effect of Group, *F*(1, 79) < 0.01, *p* = 0.99 (Figure 2A, Table S1).

Regarding MS duration, a significant main effect of Group was present, *F*(1, 79) = 12.58, *p* < 0.001, *η* ^2^ = 0.02, along with a significant Group × State interaction, *F*(3, 237) = 14.18, *p* < 0.001, *η* ^2^ = 0.13. Post hoc analyses showed that the duration of MS D was significantly reduced in the ACAD group compared to the HC group (*p* < 0.001), with no significant differences observed in other MSs (ps > 0.22). The main effect of State was not significant, *F*(3, 237) = 2.17, *p* = 0.09 (Figure 2B, Table S1).

For the GFP, there was a significant main effect of State, *F*(3, 237) = 2.69, *p* = 0.05, *η* ^2^ = 0.001, as well as a significant main effect of Group, *F*(1, 79) = 4.97, *p* < 0.03, *η* ^2^ = 0.06, and a significant Group × State interaction, *F*(3, 237) = 25.35, *p* < 0.001, *η* ^2^ = 0.01. Simple effects analyses indicated that the GFP of MS4 was significantly lower in the ACAD group compared to the HC group (*p* < 0.01), with no significant differences observed in the other states (ps > 0.35); (Figure 2C, Table S1).

Analysis of occurrence revealed that a significant main effect of Group was observed, *F*(1, 79) = 16.70, *p* < 0.001, *η* ^2^ = 0.06, as well as a significant Group × State interaction, *F*(3, 237) = 7.54, *p* < 0.001, *η* ^2^ = 0.06. Simple effects analysis showed that the occurrence of MS C was significantly higher in the ACAD group than in the HC group (*p* < 0.001). Additionally, the occurrence of MS B was marginally higher in the ACAD group compared to the HC group (*p* = 0.06). The main effect of State was not significant, *F*(3, 237) = 1.56, *p* = 0.20 (Figure 2D, Table S1).

**Figure 3:**
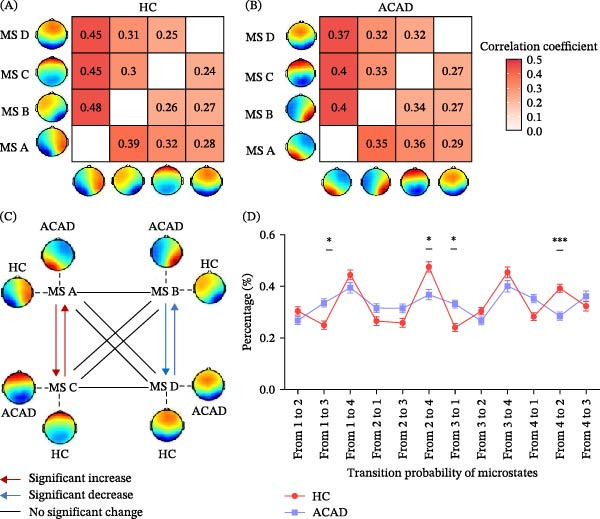
Microstate transition analysis between adolescents with comorbid anxiety and depression (ACAD) and HC (healthy control) groups. (A, B) Transition probability matrices showing correlations between microstate classes (MS A–D) for HC (A) and ACAD (B) groups. Color intensity represents correlation coefficients ranging from 0.0 to 0.5, with warmer colors indicating stronger correlations. Circular topographic maps illustrate the spatial distribution of each microstate class. (C) Network diagram depicting significant changes in microstate transitions between groups. Red lines indicate significant increases, blue lines represent significant decreases, and black lines show no significant changes in TPs from HC to ACAD groups. (D) Detailed comparison of TPs between all microstate pairs. Red circles and lines represent HC group data, while blue squares and lines represent ACAD group data. Error bars indicate SEM. Statistical significance levels are marked as  ^∗^ *p* < 0.05 and  ^∗∗∗^ *p* < 0.001.

Transition probability analyses revealed a significant main effect of State, *F*(3, 237) = 2.02, *p* = 0.02, *η* ^2^ = 0.02. A significant Group × State interaction was observed, *F*(3, 237) = 7.97, *p* < 0.001, *η* ^2^ = 0.09. Subsequent simple effects analyses indicated that the TPs from MS A to MS C and from MS C to MS A were significantly higher in the ACAD group compared to the HC group (*p* = 0.05 and *p* = 0.03, respectively). Conversely, transition probabilities from MS B to MS D and from MS D to MS B were significantly lower in the ACAD group than in the HC group (*p* = 0.03 and *p* < 0.001, respectively). The main effect of Group was not significant, *F*(1, 79) < 0.01, *p* = 0.98 (Figure 3, Table S2).

### 3.2. FC Results

**Figure 4:**
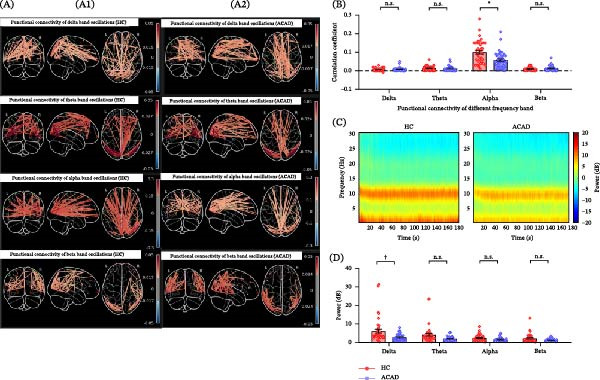
Functional connectivity and time‐frequency analysis in healthy controls (HCs) and adolescents with comorbid anxiety and depression (ACAD). (A) Visualization of functional connectivity patterns for alpha, beta, delta, and theta band oscillations. (A1) displays connectivity maps for the HC group and (A2) for the ACAD group. (B) Bar graph summarizing the correlation coefficient of functional connectivity across different frequency bands (delta, theta, alpha, and beta). Individual data points for both the HC and ACAD groups are overlaid. Statistical significance is indicated by asterisks ( ^∗^ *p* < 0.05) or “n.s.” indicates not significant. (C) Time‐frequency plots illustrating the PSD of brain oscillations for HC and ACAD groups. (D) Bar graph illustrating the PSD of EEG signals across different frequency bands (delta, theta, alpha, and beta) for both HC and ACAD groups. Individual data points are plotted, the “†” indicates *p* < 0.1, and “n.s.” denotes nonsignificant differences. Error bars represent standard error of the mean (SEM).

The FC analysis revealed a significant main effect of Frequency band, *F*(3, 237) = 15.85, *p* < 0.001, *η* ^2^ = 0.11. A significant Group × Frequency band interaction was observed, *F*(3, 237) = 9.64, *p* < 0.001, *η* ^2^ = 0.07. Follow‐up simple effects analyses indicated that FC in the alpha band was significantly lower in the ACAD group compared to the HC group (*p* = 0.04). No significant group differences were found in the FC of the other frequency bands. The main effect of Group was not significant, *F*(1, 79) < 0.01, *p* = 0.98 (Figure 4A,B, Table S3).

### 3.3. Time‐Frequency Results

The time‐frequency analysis indicated that a significant Frequency band × Group interaction was observed, *F*(3, 237) = 5.51, *p* < 0.001, *η* ^2^ = 0.02. Follow‐up simple effects analyses revealed that delta band power was marginally lower in the ACAD group compared to the HC group (*p* = 0.07). No other significant group differences were found (ps > 0.14). The main effects of Frequency band, *F*(3, 237) = 1.41, *p* = 0.24, and Group, *F*(1, 79) = 0.04, *p* = 0.85, were not significant (Figure 4C,D, Table S4).

### 3.4. SVM Classification Results

**Figure 5:**
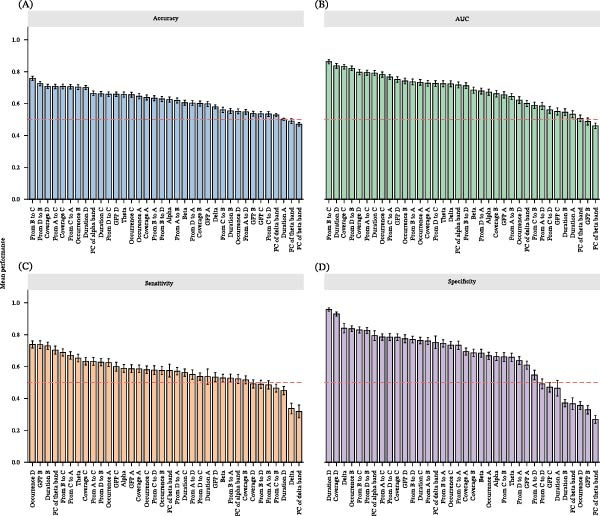
Comprehensive evaluation of single‐feature SVM classification performance for discriminating adolescents with comorbid anxiety and depression (ACAD) from healthy controls (HC). Four parts display key performance metrics: classification accuracy (A), area under the receiver operating characteristic curve (AUC, B), sensitivity (C), and specificity (D). Each bar represents a distinct EEG feature derived from microstate parameters (e. g., occurrence, duration, coverage, and intermicrostate transition probabilities), spectral power measures (delta, theta, alpha, and beta bands), or functional connectivity (FC) of specific frequency bands. Features are ranked in descending order of mean performance from left to right within each panel. Error bars indicate standard deviation across cross‐validation folds. The dashed horizontal line at 0.5 represents chance‐level performance.

The single‐feature SVM analysis estimated the independent discriminative value of each individual MS feature when evaluated in isolation. Among all individual MS features, the best‐performing single‐feature model achieved an accuracy of 75% (SD = 0.14) and an AUC of 0.86 (SD = 0.13), with the transition probability from MS B to MS C identified as the most discriminative individual feature, highlighting the critical role of specific inter‐MS dynamic transition patterns in classification performance. Sensitivity analysis indicated that the occurrence of MS D served as the most important feature for enhancing model sensitivity, achieving a sensitivity of 74% (SD = 0.22), which reflects the advantage of this feature in correctly identifying target categories. Specificity analysis demonstrated that the duration of MS D was the key determinant of model specificity, reaching a remarkably high specificity of 96% (SD = 0.10), suggesting the significant efficacy of this feature in reducing false positives (Figure 5). These results reflect each feature’s marginal, context‐free discriminative capacity and are intended to guide feature domain prioritization. Permutation testing confirmed that the classification performance of the best univariate MS SVM model (accuracy = 0.75, AUC = 0.86, sensitivity = 0.74, specificity = 0.96) significantly exceeded chance level (accuracy: permutation *p* < 0.001; AUC: permutation *p* < 0.001; sensitivity: permutation *p* = 0.04; specificity: permutation *p* < 0.001; 1000 permutations; Figure S1). These findings highlight the significant role of distinct MS characteristics in discriminating between groups.

### 3.5. MV‐SVM Classification Results

To isolate the contribution of multivariate feature integration from that of PCA‐based dimensionality reduction, an SVM classifier was trained on all MS parameters simultaneously without PCA. The MV‐SVM achieved an accuracy of 0.95 (SD = 0.07), an AUC of 0.98 (SD = 0.04), a sensitivity of 0.95 (SD = 0.11), and a specificity of 0.96 (SD = 0.11), which is higher than single‐feature SVM, demonstrating that multivariate integration of MS features —independent of any dimensionality reduction —is the primary driver of classification performance improvement.

### 3.6. PCA –SVM Classification Results

PCA was applied separately to each feature domain (MS parameters, FC metrics, and time‐frequency features) rather than to a combined feature matrix. This domain‐specific approach was adopted because the three feature sets are derived from distinct neurophysiological processes and differ substantially in scale and units. Applying a unified PCA across all domains would risk confounding the variance structure of heterogeneous feature types and would preclude meaningful comparison of the independent discriminative contribution of each domain, which isthe central objective of this study. For each domain, three PCs were retained based on scree plot elbow analysis and a cumulative variance threshold of >90%. After performing PCA separately on MS parameters, FC metrics, and time‐frequency features, the classification performance for distinguishing the ACAD group from HC was assessed (Table 2). MS parameters exhibited outstanding discriminatory power, achieving an accuracy of 0.94 (SD = 0.08), an AUC of 0.98 (SD = 0.04), as well as balanced sensitivity (0.93, SD = 0.13) and specificity (0.95, SD = 0.11). In contrast, FC features demonstrated moderate classification performance, with an accuracy of 0.63 (SD = 0.15) and an AUC of 0.72 (SD = 0.18). Notably, the specificity (0.82, SD = 0.19) was substantially higher than the sensitivity (0.46, SD = 0.25), suggesting a bias toward correctly identifying true negatives. Time‐frequency features also showed moderate discriminative ability, with an accuracy of 0.63 (SD = 0.14), an AUC of 0.70 (SD = 0.19), and relatively balanced sensitivity (0.61, SD = 0.26) and specificity (0.64, SD = 0.25), indicating comparable performance in identifying both positive and negative cases, despite moderate variability. Permutation testing further confirmed that the PCA –SVM MS model’s performance was statistically significant above chance (accuracy: permutation *p* < 0.001; AUC: permutation *p* < 0.001; sensitivity: permutation *p* < 0.001; specificity: permutation *p* < 0.02; 1000 permutations; Figure S2), providing strong evidence that the high classification accuracy reflects genuine neurophysiological group differences.

**Table 2:** Classification performance comparison of single‐feature SVM, multivariate SVM, and PCA –SVM models based on microstate features.

Model	Accuracy	AUC	Sensitivity	Specificity
Single‐feature SVM	0.75	0.86	0.74	0.96
MV‐SVM (no PCA)	0.95	0.98	0.95	0.96
PCA–SVM	0.94	0.98	0.93	0.95

Abbreviations: AUC, area under the receiver operating characteristic curve; MV ‐SVM, multivariate SVM trained on all microstate features simultaneously without dimensionality reduction; PCA, principal component analysis; SVM, support vector machine.

## 4. Discussion

This study sought to identify reliable neurophysiological markers capable of distinguishing adolescents with high levels of anxiety and depression. EEG MS analyses revealed an increase in MS C and a decrease in MS D, enhanced transition dynamics between MS A and MS C, alongside a reduced transition frequency between MS B and MS D in ACAD. The ACAD group also showed a trend toward lower delta‐band power and reduced alpha‐band FC. Notably, among all examined features, EEG MS parameters exhibited the most robust discriminative capacity, significantly outperforming both FC and time –frequency characteristics. These results highlight the potential of EEG MS analysis as a particularly sensitive approach for ACAD.

### 4.1. Neural Oscillations and Functional Connectivity Characteristics of ACAD

Reduction in delta band power in the comorbid group than controls. Given that delta oscillations are associated with motivational states triggered by salient stimuli such as rewards and dangers [46], this reduction may reflect the anhedonia and motivational deficits commonly observed in ACAD. Lower delta power has been interpreted as a potential marker of brain immaturity relative to that of adults [47]. Therefore, the current finding also suggests delayed brain development of ACAD.

We also identified a notable decrease in global alpha‐band FC in the ACAD. Alpha‐band connectivity is a critical index of brain maturation as its enhancement with age facilitates the efficient integration of information across large‐scale neural networks [47]. Aligns with previous research in anxiety disorders [57], reduced alpha FC supports the hypothesis of developmental lag. Collectively, these findings in both foundational oscillations and network‐level communication support the hypothesis that the neurophysiological profile of ACAD may be characterized by a significant delay in neuromaturational trajectories, potentially induced or exacerbated by the underlying psychopathology.

### 4.2. MS Characteristics of ACAD

Importantly, we observed a robust pattern of MS abnormalities in the ACAD. Specifically, higher occurrence and coverage of MS C alongside reduced duration and coverage of MS D. Reduced MS D duration has been reported in depression, social anxiety, and GAD, suggesting a transdiagnostic vulnerability signal [23, 24, 58]. Reduced MS D coverage has been consistently observed in depression and social anxiety [23, 24, 58]. In our adolescent cohort, MS D coverage emerged as a strong discriminative parameter for comorbidity, indicating its potential as a sensitive and generalizable index of neural alterations, even in the adolescent population.

Concurrently, the increased occurrence and coverage of MS C point to another critical facet of this comorbidity’s neurophysiological signature. While our findings partially align with reports in other psychiatric populations [23, 24], the literature on MS C remains heterogeneous, with inconsistent or null findings [25]. This variability might be from the sample characteristics or disease severity. Therefore, we propose that it is not the alteration of MS C or MS D in isolation, but rather the simultaneous increase in MS C and decrease in MS D that constitutes a highly specific and robust biomarker for ACAD. This dynamic interplay likely reflects a critical neural imbalance: a disproportionate allocation of neural resources toward self‐referential and salience processing (MS C‐related networks), contributing to sustained negative rumination, while executive attention control (MS D‐related networks) is concurrently compromised, resulting in diminished cognitive flexibility. Although a significant main effect of the MS class on GFP was observed, the small effect size reflects the intrinsic amplitude homogeneity across MS topographies [42, 59], with GFP values remaining within a narrow physiological range across these classes. Importantly, this underscores that the core neurobiological mechanisms and the high classification performance observed in adolescents with ACAD are driven by disrupted temporal dynamics rather than alterations in GFP.

Beyond alterations in MS parameters, our findings on TPs reveal a profound dysregulation in the dynamic switching between neural networks in adolescents with anxiety –depression comorbidity. We identified a dual signature of altered dynamics: an increased transition rate between MS A from the auditory network and MS C from the saliency/default mode network (DMN) and a decreased rate between MS B from the visual network and MS D from the attention control network [18].

The elevated TP between MS A and MS C suggests a functional hyper‐coupling between sensory processing and self‐referential thought. MS C, associated with the salience and DMNs, is crucial for self‐referential processing and cognitive control [18, 60]. Critically, hyperactivity and hypercoherence within the DMN are linked to ruminative symptoms in MDD with comorbid anxiety [61]. Speculatively, our finding of increased MS A↔MS C transitions thus delineates a potential neural mechanism for this rumination: a state where external auditory stimuli (MS A) may excessively and rapidly trigger internal, self ‐focused, and often negative thought patterns (MS C), creating a vicious cycle of maladaptive processing [23].

Conversely, the reduced TP between MS B and MS D points to an impaired dynamic coordination between sensory intake and top‐down cognitive control. This hypoconnectivity implies a weakened ability to flexibly shift from processing visual information (MS B) to engaging attentional and executive resources (MS D). This finding is consistent with previous reports in both anxiety [62] and depression [63], suggesting that it is a robust marker of internalizing disorders. Notably, the normalization of this specific transition (D to B) has been linked to successful selective serotonin reuptake inhibitor treatment in MDD, highlighting its clinical relevance and potential as a therapeutic biomarker [63].

Taken together, these opposing shifts in MS transitions paint a compelling picture of a dynamic imbalance in cognitive resource allocation. The neural landscape in these adolescents appears biased toward an overactive pathway linking sensory input to internal rumination (MS A↔MS C), concurrent with a weakened pathway for engaging executive control to modulate cognition (MS B↔MS D). This provides a compelling neurophysiological model for the core clinical phenotype: a heightened vulnerability to being captured by negative thoughts, coupled with a diminished capacity to disengage and flexibly reallocate attentional resources.

### 4.3. Optimal Features for Identifying ACD

Although EEG MSs have been proposed as biomarkers for psychiatric disorders [63, 64], ACAD has been largely overlooked. Addressing this gap, we found that single‐feature classifiers based on MSs parameters outperformed those using FC or time‐frequency features across all evaluation metrics (accuracy, sensitivity, specificity, and AUC). This suggests that aberrant brain dynamics provide greater discriminative power for this population. Univariate feature evaluation revealed that the transition probability from MS B to MS C carried the highest independent discriminative value among individual MS features (accuracy = 75% and AUC = 0.86), identifying MS transition dynamics as the most informative feature domain for subsequent multivariate modeling. Importantly, these univariate results reflect each feature’s marginal predictive capacity evaluated in isolation and are not directly comparable to multivariate classification performance.

Converging evidence also indicates that relying on isolated MS features can be limiting. For example, a study attempting to classify obsessive –compulsive disorder with single MSs metrics reported poor performance and argued that combining MS features yields a more reliable feature set for machine learning [65]. To rigorously disentangle the contributions of multivariate feature integration and PCA‐based dimensionality reduction, we compared three classification conditions. The MV‐SVM model, which included all MS features without PCA, achieved a better classification performance than the single‐feature SVM. Because PCA was absent in both conditions and only the number of included features varied, this comparison demonstrates that multivariate integration of MS parameters, rather than PCA, was the primary source of improved classification performance. In contrast, the PCA‐based SVM did not show a substantial improvement over the MV‐SVM as this comparison held the feature set constant and varied only whether PCA was applied. These results indicate that PCA contributed only marginal additional performance gains while nevertheless providing meaningful methodological benefits. Specifically, by reducing the correlated MS feature set to three uncorrelated PCs explaining more than 90% of the variance, PCA mitigated multicollinearity, reduced model complexity, and lowered the risk of overfitting in this small clinical sample, thereby enhancing the potential generalisability of the classification model to independent cohorts. These results support the view that integrated alterations in MS dynamics, rather than state‐specific metrics considered in isolation, constitute the most promising feature space for differentiating ACAD.

From a developmental neuroscience perspective, adolescent brain functional maturation follows a developmental trajectory “from local to distributed processing,” facilitating enhanced efficiency in large‐scale information integration and marking the brain’s gradual transition toward global processing modes [66]. Adolescent brain dysfunction is not confined to isolated alterations in individual brain regions but manifests as a systematic reorganization of large‐scale brain network connectivity [67]. Therefore, integrated MS features that reflect global brain dynamic changes are better suited to capture the complex pathological mechanisms underlying ACAD. This theoretical framework provides a neurobiological explanation for the comprehensive improvements achieved by MS feature integration strategies across all SVM evaluation metrics, emphasizing the central role of global brain network dynamics in adolescent psychopathology identification.

### 4.4. Significance and Contributions

A pivotal contribution of this study lies in its systematic comparison of multimodal neurophysiological features to identify robust biomarkers for ACAD. Our findings unequivocally demonstrate that MS dynamics not only outperform but substantially surpass traditional FC and time‐frequency metrics in the classification performance. Our work provides a crucial methodological validation that for the specific clinical population, brain dynamics offers superior discriminative power compared to static or spectral measures.

The significance of this finding; however, extends beyond a mere methodological preference. It reveals a fundamental insight into the nature of the disorder itself. MSs excel precisely because they capture the core pathophysiology of this comorbidity: a multifaceted and dynamic system‐level dysregulation. This is manifested in two key ways identified by our study: first, the dysregulated interplay between MS C and MS D may reflect a persistent struggle between internal, self‐referential thought and external, goal‐directed attention. Second, the global alterations in network switching dynamics reflect an entrenched, system‐wide aberrant mode of brain function rather than a localized deficit.

Therefore, this study offers two primary contributions. First, it establishes an integrated profile of MS dynamics as a more potent and neurophysiologically relevant biomarker for ACAD. Second, and more importantly, it provides a theoretically grounded framework that reframes this developmental psychopathology not as a static condition but as a disorder of whole‐brain dynamic coordination. This work paves the way for developing more objective, brain‐based assessments to aid in the diagnosis and monitoring of at‐risk adolescents.

## 5. Limitations

Although this study provides valuable insights into the neurophysiological mechanisms of ACAD, several limitations should be considered.

First, despite our efforts to identify reliable biomarkers, the findings may have been influenced by multiple confounding variables, such as illness duration [68], medication status [69], and differences in measurement instruments [9]. Future studies should employ larger and more homogeneous samples to systematically examine the impact of these factors and verify the robustness and reproducibility of the present results.

Second, it remains uncertain whether task‐based paradigms offer higher precision than resting‐state assessments for distinguishing anxiety from depression. This question is particularly relevant given the known cognitive –emotional biases —anxiety is associated with heightened threat sensitivity, whereas depression is linked to reduced reward responsiveness [70]. Prior MS studies have shown that anxiety can impair monitoring processes [71], especially under conditions of unpredictability where conflict control is further compromised [72]. Clarifying this methodological issue could enhance the specificity and translational potential of biomarker identification.

Third, although our results demonstrate promising classification performance, further longitudinal validation is required to confirm the clinical utility of the identified biomarkers, particularly in predicting symptom exacerbation and monitoring recovery trajectories. Establishing their prognostic value could facilitate early detection, risk stratification, and targeted intervention in adolescent populations.

## 6. Conclusion

This study demonstrates that ACAD is characterized by a multidimensional profile of large‐scale brain network dysfunction, with EEG MS dynamics emerging as the most sensitive biomarkers. The combined pattern of increased MS C and decreased MS D, alongside aberrant TPs, reflects a fundamental imbalance in cognitive resource allocation. By systematically comparing multimodal neurophysiological features, our results reveal that the primary source of classification improvement is the multivariate integration of MS parameters, while PCA contributes additional methodological benefits in terms of model parsimony and generalizability. These findings not only validate MS dynamics as potential biomarkers but also offer a neurobiological framework for early identification, risk stratification, and targeted intervention in adolescent internalizing disorders.

## Funding

This research was supported by the National Natural Science Foundation of China (Grant 32371104), STI 2030‐Major Projects (Grant 2021ZD0200500), Project (PT‐2024‐06B) from the National Human Genetic Resources Sharing Service Platform (Grant 2005DKA21300), the Fundamental Research Funds for the Central Universities (Grant 2243300005), the Major Project of National Social Science Foundation (Grant 20&ZD153), Shenzhen‐Hong Kong Institute of Brain Science – Shenzhen Fundamental Research Institutions (Grant 2025SHIBS0003), MOE (Ministry of Education in China) Project of Humanities and Social Sciences (Grant 24YJC190009), Guangdong Province Philosophy and Social Sciences Planning Youth Project (Grant GD24YXL01), Guangdong Province Basic and Applied Research Fund for Regional Collaborative Projects (Grant 2025A1515110116), and Guangzhou University Graduate Student Innovation Ability Cultivation Basic Research Project (Grants JCCX2025020 and JCCX2026023).

## Ethics Statement

The study was approved by the Ethics Committee of Guangzhou University (number 2023052).

## Consent

Written informed consent was obtained from all the participants in this study.

## Conflicts of Interest

The authors declare no conflicts of interest.

## Supporting Information

Additional supporting information can be found online in the Supporting Information section.

## Supporting information


**Supporting Information 1** Table S1 reports descriptive statistics of duration, coverage, occurrence, and global field power (GFP) in healthy controls (HC) and adolescents with anxiety –depression comorbidity (ACAD). Table S2 presents descriptive statistics of microstate transition probabilities in HC and ACAD. Table S3 provides descriptive statistics of functional connectivity across delta, theta, beta, and alpha bands in HC and ACAD. Table S4 shows descriptive statistics of frequency power across delta, theta, beta, and alpha bands in both groups.

**Supporting Information 2** Figure S1 illustrates the permutation test results of the univariate microstate SVM classification, including null distributions of accuracy, AUC, sensitivity, and specificity, with observed performance indicated. Figure S2 presents the permutation test results of the multivariate PCA –SVM microstate model, showing null distributions for the same performance metrics and highlighting statistically significant above‐chance classification.

## Data Availability

The data that support the findings of this study are available from the corresponding author upon reasonable request.
