# Supplementary material for: Disrupted Global Brain Dynamics in Adolescents With Comorbid Anxiety and Depression: Neural Mechanisms and Classification Based on EEG Microstates
Source: Depress Anxiety. 2026 Jul 15;2026:4012249. doi: 10.1155/da/4012249 (PMC13373315; doi:10.1155/da/4012249)
Supplement: Supplementary file 1 — Supporting Information 1 Table S1 reports descriptive statistics of duration, coverage, occurrence, and global field power (GFP) in healthy controls (HC) and adolescents with anxiety –depression comorbidity (ACAD). Table S2 presents descriptive statistics of microstate transition probabilities in HC and ACAD. Table S3 provides descriptive statistics of functional connectivity across delta, theta, beta, and alpha bands in HC and ACAD. Table S4 shows descriptive statistics of frequency power across delta, theta, beta, and alpha bands in both groups. [file DA-2026-4012249-s002.docx]

|  | HC | | | |  | ACAD | | | |
| --- | --- | --- | --- | --- | --- | --- | --- | --- | --- |
|  | Duration | Coverage | Occurrence | GFP |  | Duration | Coverage | Occurrence | GFP |
| MS A | 70.75 ± 1.95 | 0.17 ± 0.01 | 2.30 ± 0.14 | 6.72 ± 0.37 |  | 69.76 ± 1.26 | 0.19 ± 0.01 | 2.69 ± 0.10 | 5.57 ± 1.88 |
| MS B | 83.54 ± 2.69 | 0.25 ± 0.02 | 2.83 ± 0.14 | 7.08 ± 0.37 |  | 94.34 ± 5.09 | 0.33 ± 0.02 | 3.38 ± 0.07 | 6.39 ± 0.38 |
| MS C | 72.45 ± 2.81 | 0.18 ± 0.02 | 2.35 ± 0.15 | 6.98 ± 0.40 |  | 75.76 ± 1.78 | 0.24 ± 0.01 | 3.18 ± 0.11 | 6.07 ± 0.33 |
| MS D | 121.63 ± 8.86 | 0.40 ± 0.03 | 3.32 ± 0.07 | 7.92 ± 0.42 |  | 75.39 ± 1.88 | 0.24 ± 0.01 | 3.13 ± 0.11 | 5.81 ± 0.30 |

**Supplementary Tables**

Table S1 Descriptive Statistics of Duration, Coverage, Occurrence, GFP in HC and ACAD (*M* ± *SE*)

Abbreviations: MS means microstate; HC means healthy controls; ACAD means adolescents with anxiety–depression comorbidity. The same applies below.

Table S2 Descriptive Statistics of transition probabilities in HC and ACAD (*M* ± *SE*)

|  |  |  | To | | | |
| --- | --- | --- | --- | --- | --- | --- |
| Group | Frome |  | MS A | MS B | MS C | MS D |
| HC | MS A |  | - | 0.31 ± 0.02 | 0.25 ± 0.02 | 0.45 ± 0.02 |
|  | MS B |  | 0.23 ± 0.02 | - | 0.26 ± 0.02 | 0.48 ± 0.02 |
|  | MS C |  | 0.24 ± 0.01 | 0.30 ± 0.02 | - | 0.46 ± 0.02 |
|  | MS D |  | 0.28 ± 0.02 | 0.39 ± 0.02 | 0.32 ± 0.02 | - |
| ACAD | MS A |  | - | 0.27 ± 0.01 | 0.34 ± 0.01 | 0.40 ± 0.02 |
|  | MS B |  | 0.32 ± 0.01 | - | 0.32 ± 0.07 | 0.37 ± 0.02 |
|  | MS C |  | 0.33 ± 0.02 | 0.27 ± 0.01 | - | 0.40 ± 0.02 |
|  | MS D |  | 0.35 ± 0.01 | 0.29 ± 0.01 | 0.36 ± 0.01 | - |

Table S3 Descriptive Statistics of functional connectivity in HC and ACAD (*M* ± *SE*)

|  | FC of delta band | FC of theta band | FC of beta band | FC of alpha band |
| --- | --- | --- | --- | --- |
| HC | 0.008 ± 0.001 | 0.014 ± 0.002 | 0.101 ± 0.009 | 0.011 ± 0.008 |
| ACAD | 0.009 ± 0.002 | 0.012 ± 0.002 | 0.059 ± 0.007 | 0.013 ± 0.002 |

Abbreviations: FC means functional connectivity.

Table S4 Descriptive Statistics of frequency power in HC and ACAD (*M* ± *SE*)

|  | Power of delta band | Power of theta band | Power of beta band | Power of alpha band |
| --- | --- | --- | --- | --- |
| HC | 6.09 ± 1.02 | 4.10 ± 0.73 | 2.12 ± 0.34 | 2.40 ± 0.26 |
| ACAD | 2.78 ± 0.28 | 2.01 ± 0.20 | 1.22 ± 0.13 | 1.59 ± 0.18 |
