# Supplementary material for: Disrupted Global Brain Dynamics in Adolescents With Comorbid Anxiety and Depression: Neural Mechanisms and Classification Based on EEG Microstates
Source: Depress Anxiety. 2026 Jul 15;2026:4012249. doi: 10.1155/da/4012249 (PMC13373315; doi:10.1155/da/4012249)
Supplement: Supplementary file 2 — Supporting Information 2 Figure S1 illustrates the permutation test results of the univariate microstate SVM classification, including null distributions of accuracy, AUC, sensitivity, and specificity, with observed performance indicated. Figure S2 presents the permutation test results of the multivariate PCA –SVM microstate model, showing null distributions for the same performance metrics and highlighting statistically significant above‐chance classification. [file DA-2026-4012249-s001.docx]

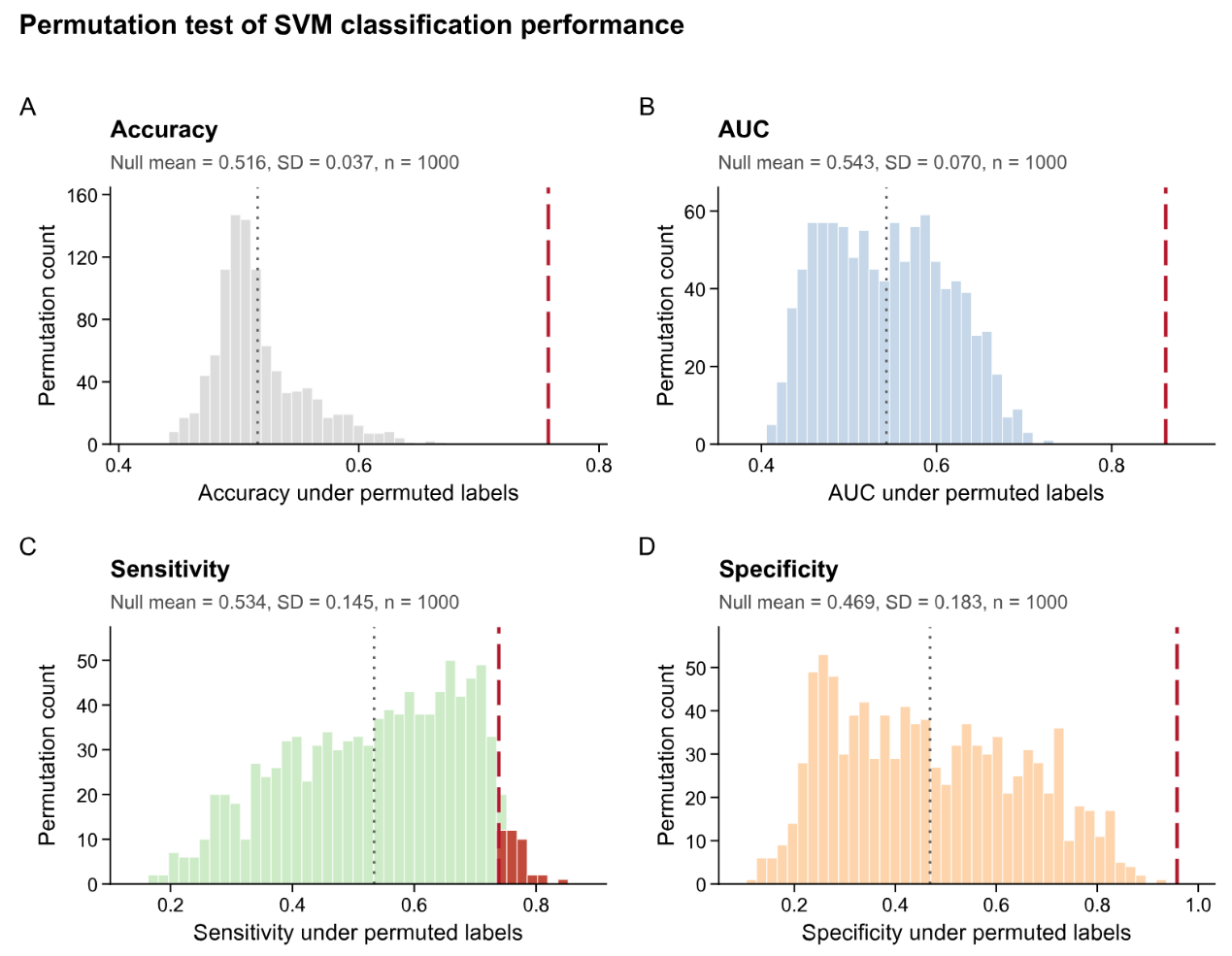
**Figure S1. Permutation test of SVM classification performance.** Null distributions of (A) accuracy, (B) AUC, (C) sensitivity, and (D) specificity derived from 1000 label permutations. Each histogram represents the distribution of the corresponding performance metric obtained when class labels were randomly shuffled while the feature matrix was held constant. Dotted vertical lines indicate the null distribution mean. Red dashed vertical lines indicate the observed classification performance of the best-performing univariate microstate SVM model. In panel C, red-shaded bars indicate permuted values that met or exceeded the observed sensitivity, illustrating the proportion of permutations contributing to the empirical *p*-value. All four observed metrics fell in the extreme tail of their respective null distributions, confirming that classification performance was statistically significant above chance level (all permutation *p* < 0.05).


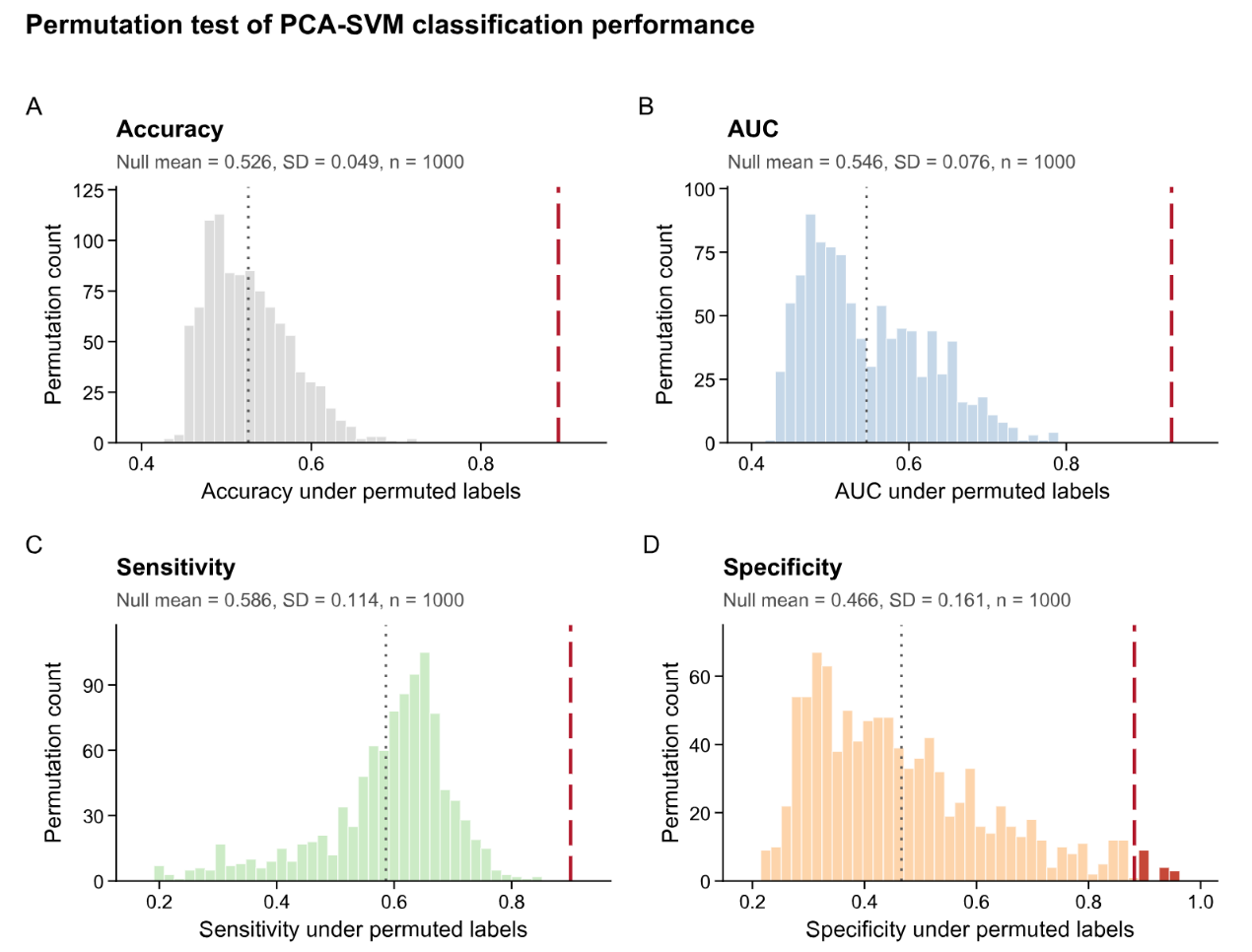


**Figure S2. Permutation test of PCA–SVM classification performance.** As in the preceding figure, but for the multivariate PCA–SVM microstate model. Null distributions of (A) accuracy, (B) AUC, (C) sensitivity, and (D) specificity are shown. In panel D, red-shaded bars indicate permuted specificity values meeting or exceeding the observed value. All four observed metrics were located in the extreme tail of their respective null distributions, confirming statistically significant above-chance classification performance (all permutation *p* < 0.05).
